# Male Anabolic Androgenic Steroid Users with Personality Disorders Report More Aggressive Feelings, Suicidal Thoughts, and Criminality

**DOI:** 10.3390/medicina56060265

**Published:** 2020-05-28

**Authors:** Annica Börjesson, Christian Möller, Anders Hagelin, Veronica Vicente, Anders Rane, Mikael Lehtihet, Marja-Liisa Dahl, Nina Gårevik, Lena Ekström

**Affiliations:** 1 Division of Clinical Pharmacology, Department of Laboratory Medicine, Karolinska Institutet, Karolinska University Hospital, Huddinge, 141 86 Stockholm, Sweden; anders.hagelin@sll.se (A.H.); anders.rane@ki.se (A.R.); marja-liisa.dahl@ki.se (M.-L.D.); nina.garevik@ki.se (N.G.); lena.ekstrom@ki.se (L.E.); 2 Centre for Ethics, Law and Mental Health, Institute of Neuroscience and Physiology, Sahlgrenska Academy, University of Gothenburg, 405 30 Gothenburg, Sweden; christian.moller@rmv.se; 3 Psychiatric Clinic, Sahlgrenska University Hospital, 413 45 Gothenburg, Sweden; 4 Department of Forensic Psychiatry, National Board of Forensic Medicine, 422 49 Gothenburg, Sweden; 5 The Ambulance Medical Service in Stockholm (AISAB), Academic EMS, 121 63 Stockholm, Sweden; veronica.vicente@ki.se; 6 Department of Clinical Science and Education, Södersjukhuset, Karolinska Institutet, 171 77 Stockholm, Sweden; 7 Department of Medicine, Karolinska Institutet, S:t Görans Hospital, 122 19 Stockholm, Sweden; mikael.lehtihet@ki.se; 8 Department of Neurobiology Care Sciences and Society, Karolinska Institutet, 171 77 Stockholm, Sweden

**Keywords:** anabolic androgenic steroids (AAS), adverse effects of psychiatric disorders, structured clinical interview diagnosis, comprehensive psychopathological rating scale, personality disorders

## Abstract

*Background and objectives:* Anabolic androgenic steroids (AAS) are mainly used for aesthetic and performance-enhancing reasons. Their use is a growing public health problem and concern for society because of their adverse effects. The primary aim of this study was to identify psychiatric and personality disorders and to measure anxiety and depression in AAS users. *Materials and Methods:* Fifty-six males who actively contacted the Anti-Doping Hot-Line and wished to stop using AAS were included. Structured Clinical Interviews Diagnosis-I and -II were used to diagnose psychiatric and personality disorders. The Brief Scale for Anxiety and Montgomery Asberg Depression Rating Scale (subscales from the Comprehensive Psychopathological Rating Scale) were used to measure changes in anxiety and depression. Structured Clinical Interviews Diagnosis-I and -II were performed at one time point. Anxiety and depression were measured at inclusion and after six months. Urine samples were collected for an analysis of AAS and drugs of abuse. *Results:* All participants reported some adverse effects that they associated with AAS use. In total, 56% and 52% of the cohort fulfilled the criteria for Structured Clinical Interviews Diagnosis-I and -II diagnoses, respectively. A significantly increased risk of reporting aggressive feelings/behaviors (Odds Ratio (OR) = 4.9; Confidence Interval (CI) 0.99–25, *p* = 0.04), suicidal thoughts/attempts (OR = 4.6, CI 95; 0.99–21, *p* = 0.04) and criminality (OR = 6.5, CI 1–39, *p* = 0.03) was found among individuals with AAS use fulfilling the criteria for personality disorders compared with those without such AAS use. The Brief Scale for Anxiety score decreased from the median of 15 at inclusion to 10 at the follow-up visit six months later (*p* = 0.01, *n* = 19). *Conclusions:* Our findings indicate that among individuals with AAS use, those with a personality disorder report more aggressive behaviors, suicidal thoughts/suicidal attempts, and criminality than those without a personality disorder.

## 1. Introduction

Anabolic androgenic steroids (AAS) are widely used in non-medical conditions to gain muscle mass [1]. While AAS use was previously considered to mainly be a problem in elite sports, it is now widespread in many countries and a growing public health problem and concern for society [2,3]. Many other drugs are often used in combination with AAS [4,5,6], in order to attenuate AAS-induced adverse effects [7]. Commonly self-reported somatic adverse effects of AAS in men include gynecomastia, potency problems, and acne [5,7]. Psychiatric symptoms include depression, aggressiveness, anxiety, sleeping disorders, and mood disturbances [2,8]. Structured Clinical Interview for Diagnosis (SCID)-I and -II are used to diagnose psychiatric and personality disorders (PD), respectively [9]. In studies using SCID, psychiatric symptoms associated with AAS use have been reported [10,11] and the incidence of SCID I and II diagnoses has been seen to be higher in users of Performance and Image Enhancing Drugs compared to non-users [12]. It has been suggested that the prevalence of PD is high among AAS users and that it may be a risk factor for psychiatric symptoms often associated with AAS [13]. Most of the studies on AAS-related psychiatric symptoms have been based on questionnaires or case reports, while only a few have been performed in cohorts of AAS users that have been clinically evaluated and subjected to AAS and drug testing [11].

The primary aim of the present study was to identify psychiatric symptoms and disorders by using SCID and the Comprehensive Psychopathological Rating Scale (CPRS) questionnaire. SCID was performed at one time point to be able to investigate the relationship between personality disorders and self-reported mental symptoms and behaviors. The Brief Scale for Anxiety (BSA) [14] and Montgomery Asberg Depression Rating Scale (MADRS) [15] are subscales derived from CPRS and were used to measure changes in anxiety and depression during and six months after stopping AAS use.

## 2. Materials and Methods

### 2.1. Study Design

The data in this report is from a prospective intervention study aimed at characterizing the health risks associated with AAS in male AAS users and to follow them for two years after stopping AAS use. During the entire study, detailed interviews, psychiatric evaluations, urine sampling, and physical examinations were conducted. The data collection was conducted by nurses, psychiatrists, and clinical pharmacologists at the Anti-Doping Hot-Line, Department of Clinical Pharmacology, Karolinska University Hospital, Stockholm, Sweden. The Anti-Doping Hot-Line, established in 1993, is an anonymous free telephone counseling service for people concerned about or affected by non-medical use of AAS [2]. We have previously shown that various cardiovascular and endocrine parameters improved after the cessation of AAS use in this study cohort [16,17]. In this sub-study, we report psychiatric symptoms and diagnoses based on CPRS, SCID-I, and SCID-II, and relate them to the pattern of AAS use.

**Figure 1:**
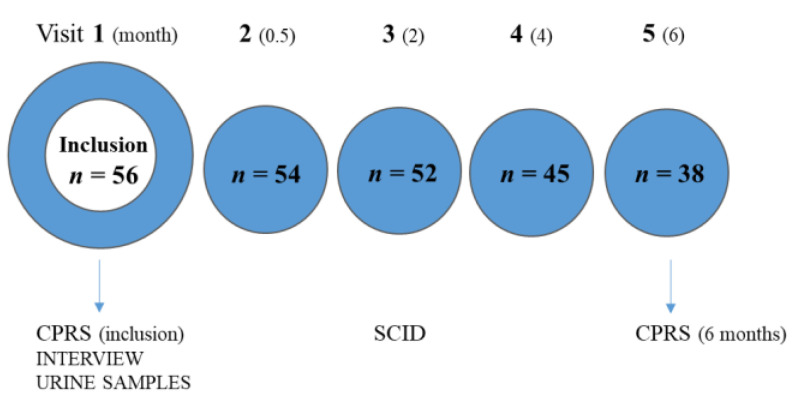
Timeline of the study, with the number of participants (= *n*) remaining in the study (within circles) at each planned visit (above the circles). CPRS = Comprehensive Psychopathological Rating Scale, SCID = Structured Clinical Interview for Diagnosis.

The study cohort consists of 56 men with the wish to stop their self-reported AAS use. The subjects were recruited from a total of about 1000 men who contacted the Anti-Doping Hot-Line between 1998 and 2002 [17]. The study was approved on 2 June 1998, ref. 186/98, by the Research Ethics Review Committee at Huddinge University Hospital, Stockholm, Sweden. The ethical standards of the Declaration of Helsinki were respected [18]. Participation commenced after oral and written informed consent was obtained. No economical remuneration was given. If deemed necessary based on the medical and/or psychiatric evaluation before or during the study, the individuals were referred to qualified medical assessment and treatment at the Psychiatric or Endocrine Departments of Karolinska University Hospital. Only fourteen (25%) of the 56 individuals completed the entire intervention study, with a follow-up time of 2 years. The timeline of this sub-study and the number of study subjects remaining in the study over time are given in Figure 1. The most common reasons for the drop outs were not turning up at follow-up visits (*n* = 16), a lack of motivation due to “good health” (*n* = 6), relapse into the abuse of AAS (*n* = 4) or drugs of abuse (*n* = 3), and not agreeing to a change of contact nurse (*n* = 3).

### 2.2. Data Collection

#### 2.2.1. Interviews

All 56 individuals were interviewed at the first visit by a single physician and an experienced nurse working at the Anti-Doping Hot-Line. A semi-structured standard questionnaire was used and the questions related to (1) demographics, (2) details about AAS use history and patterns, (3) motives for starting AAS, (4) the co-use of other doping agents and narcotic substances, (5) exercise patterns, (6) family backgrounds, and (7) any experienced adverse effects. In the analysis, the content of the interviews were compiled quantitatively [19]. The text was first read in its entirety to get familiar with the data. In the second step, the text was divided into smaller parts on a manifest level. We counted the most common words and compiled these descriptively.

#### 2.2.2. Structured Clinical Interview for Diagnosis

SCID I and II are psychiatric semi-structured interviews employed for diagnosing current or past psychiatric conditions [9]. Between visits 2 and 5, SCID I (psychiatric disorders) and SCID II (personality disorders) interviews were performed at one time point by an experienced psychiatrist (CM). Diagnoses were made using criteria according to the Diagnostic and Statistical Manual of Mental Disorders (DSM) [20]. DSM classifies mental disorders and is used as a support to improve diagnoses, treatment, and research.

#### 2.2.3. Brief Scale for Anxiety and Montgomery Asberg Depression Rating Scale

At inclusion and in month six, trained nurses conducted semi-structured interviews using CPRS [21]. CPRS assesses present psychiatric symptoms within a time frame of the last 3 days [22]. It is a clinically used rating instrument and consists of 65 items covering a wide range of psychiatric signs and symptoms. In this study, we used two of CPRS subscales, BSA [14] and MADRS [15], to be able to measure changes in anxiety and depression. Each of the subscales contains ten variables. The individual items are rated from 0 (absence of symptom) to 3 (extreme degree of the symptom). A total score of ≥11, together with at least three items rated as ≥2, strengthens the suspicion of anxiety and depression, respectively. In addition, some of the individual items must be rated to meet criteria for anxiety and depression [23].

#### 2.2.4. Urine Samples

Urine samples were collected for an analysis of AAS and drugs of abuse. Urine samples were analysed at the Drugs of Abuse Laboratory and the World Anti-Doping Agency (WADA) accredited Anti-Doping Laboratory, at the Department of Clinical Pharmacology, all at Karolinska University Hospital, Sweden. For endogenous steroids, i.e., testosterone, the traditional biomarker for testosterone doping, the testosterone/epitestosterone (T/E) ratio [24] was used. Exogenous steroids and drugs of abuse were identified following the WADA guidelines. The AAS and drugs of abuse were analyzed with gas chromatography-mass spectrometry—the routine methods at the time of the study.

#### 2.2.5. Statistical Analysis

Paired non-parametric (Wilcoxon matched-pairs signed rank test) analysis was performed to evaluate differences in MADRS and BSA scores between inclusion and the six-month follow-up. Correlation analyses between hormone levels and CPRS scores for BSA and MADRS were performed by using a Spearman Rank test. Findings at *p* ˂ 0.05 (two tailed) were considered significant.

For SCID diagnosis, a Chi-squared test (Graph Prism v5, San Diego, CA, USA) was used to analyse differences in the frequencies of self-reported psychiatric adverse effects (including aggression and suicide attempts) between individuals with and without a SCID II PD diagnosis.

## 3. Results

### 3.1. Structured Clinical Interview for Diagnosis

SCID I and II were conducted in 39 and 31 of the individuals, respectively. In total, 31 individuals completed both SCID I and II.

The criteria for one or more SCID I diagnosis was fulfilled by 22 subjects (56% of the 39 with completed SCID I) (Table 1). The most common diagnosis was depression. All four individuals with bipolar disorder experienced mania-like symptoms during periods of AAS use, especially during the early phase (weeks to a few months) of use. Mood swings, impulsivity, and increased verbal and physical aggression were noted after longer (more than three months) periods of AAS use. During the periods following the cessation of AAS use, almost all individuals experienced feelings of low self-esteem, a lack of confidence, and intense feelings of discomfort.

The criteria for one or more PDs was fulfilled by 16 (52% of the 31 with completed SCID II) individuals (Table 1). The most common diagnoses were antisocial and borderline PD. Criteria for one PD were fulfilled by eleven individuals (69%), two disorders by four (25%), and features from several PDs by one (6%). Thirteen individuals diagnosed with PD self-reported aggression (81%), ten suicidal thoughts /suicide attempts (63%), eight violence (50%), and eight criminal activities (50%) during AAS use. Individuals with a SCID II diagnosis showed a significantly increased risk of self-reporting aggressive feelings/behaviors (Odds Ratio (OR) = 4.9; Confidence Interval (CI) 0.99–25, *p* = 0.04) and suicidal attempts (OR = 4.6, CI 95; 0.99–21, *p* = 0.04) compared to those with no SCID diagnoses. When testing for different aggressive behaviors, such as self-reports of violence and criminal activities, a significantly increased risk for criminality was found among individuals with a SCID diagnosis (OR = 6.5, CI 95; 1–39, *p* = 0.03).

### 3.2. Used and Co-Used Substances and Motives for Using AAS

The characteristics of AAS use in the 56 male study subjects are given in Table 2. Comparisons of the individuals with and without PD diagnoses showed no significant differences in any of the characteristics. Notably, more individuals diagnosed with a PD started their use of AAS < 18 years of age (*n* = 7) compared with a non-PD diagnosis (*n* = 1) (*p* = 0.03). The ongoing use of narcotic agents and alcohol was more common in individuals diagnosed with PD (*p* = 0.03) than in non-PD AAS users.

At inclusion, 47 (84%) had positive urine tests for AAS (Table 3). Four (7%) had positive tests for cannabis and one (2%) for cocaine.

Most of the participants (*n* = 49, 87%) practiced stacking, i.e., the use of several different AAS agents and in cycles. Three individuals did not take AAS in regular cycles, but used them continuously. Half of the population (*n* = 28, 50%) reported to have started their AAS use with non-injectable preparations. Most of them later switched to injectable formulations, since 53 (95%) reported the ongoing use of an injectable substance. Detailed information on the AAS schedules (type of substance and daily dose) provided by the individuals are given in a Supplementary File.

Seventy-three percent (*n* = 41) used/had been using other drugs in addition to AAS. These drugs were other doping agents, medications to avoid or treat adverse effects of AAS, and drugs for fat loss (Table 4).

Dietary supplements were also commonly reported (*n* = 43, 77%). Three out of four individuals reported the use of narcotics at some point during their lifetime.

The most commonly reported reason for starting the use of AAS was to increase muscle mass and/or improve performance (*n* = 42, 75%). However, only five (9%) of the individuals competed in bodybuilding. The improvement of self-esteem (*n* = 23, 41%) and alleviation of pain (*n* = 3, 5%) were other reasons and motivations. Some individuals (*n* = 10, 18%) were influenced by a person close to them (trainers *n* = 4, father *n* = 3, and friends *n* = 3) to start using AAS. Fifty subjects (89%) reported that they were regular gym customers, and it was most common that they had started to use AAS within one year after starting strength training.

### 3.3. Family and Social Background

A large number of individuals self-reported some sort of problems during their childhood, i.e., not attending school, having abusive parents, split families, a dead parent, an eating disorder, or shoplifting. Only 15 (28%) were raised with two present parents and 23 (41%) described a bad relationship with either or both of their parents. Of the 56 individuals, suicidal thoughts and suicidal attempts during AAS use were reported by 14 and 6 individuals, respectively (in total *n* = 17, 30%). Of these, 13 (77%) reported the intake of narcotic agents at the time of suicidal thoughts/attempts. Twenty-four (43%) of the 56 individuals were engaged in “criminal” activities and 19 (34%) had been in contact with the police for assault, the peddling of AAS, theft, or robbery.

### 3.4. Adverse Effects

All participants self-reported at least one adverse effect that they related to their AAS use. One to five adverse effects were reported by 12 individuals (21%) and six to ten by 40 (71%), and the rest reported more than 10 adverse effects. Aggression was the most common self-reported psychiatric symptom during AAS use; of these individuals, 17 (42%) used drugs at the same time. Only five (9%) reported aggressive feelings prior to starting AAS use. Violence was reported by 25 individuals (45%), who described that they often ended up fighting. Violence was often directed at relatives. Only two individuals stated that they had been violent before they started with AAS. Among the individuals who reported depression, only three (5%) had experienced depressive feelings prior to their AAS use. The most commonly reported psychiatric symptoms and somatic adverse effects are given in Table 5.

### 3.5. The Brief Scale for Anxiety and Montgomery Asberg Depression Rating Scale

**Figure 2:**
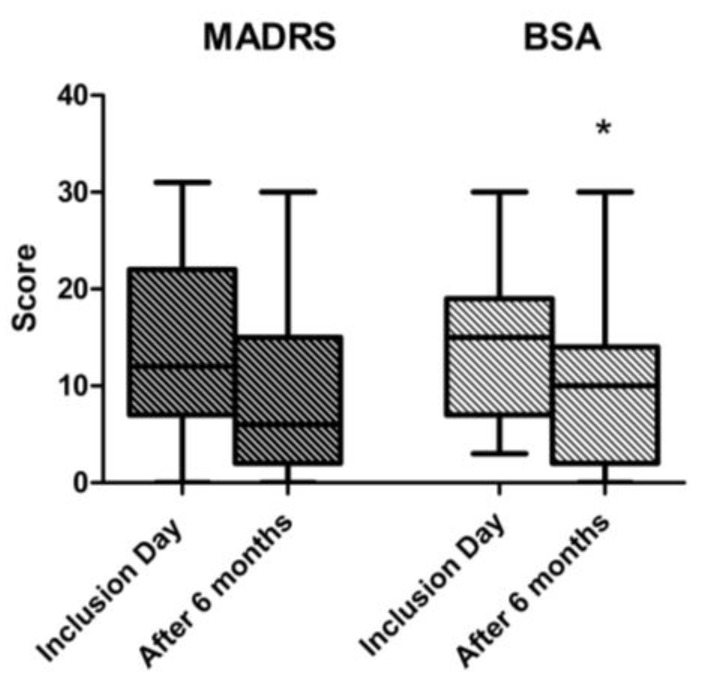
Box and whisker plots (Median, Interquartile range (IQR), and Min-Max values) of the Montgomery Asberg Depression Rating Scale (MADRS) (depression) and Brief Scale for Anxiety (BSA) (anxiety) score at inclusion and at the six-month follow-up. Paired analysis in 19 individuals (* = *p* ˂ 0.05).

CPRS was performed in 51 (91%) of the individuals on the inclusion day and in 19 (37%) at the six-month follow-up. At inclusion, 35 (69%) and 24 (47%) of the individuals rated a score ≥ 11 on the subscales for BSA and MADRS, respectively. There was a significant decrease in the BSA score from inclusion to the six-month follow-up (*p* < 0.01, Figure 2, *n* = 19).

However, according to the guidelines for the assessment of BSA and MADRS, only 13 (25%) fulfilled the criteria for an anxiety disorder and 18 (35%) for depression. At the follow-up, two met the guideline criteria for anxiety and five for depression.

There were individual items from the total CPRS questionnaire that were rated high in terms of the CPRS score. We thought this was worth noting because the individuals noted that these symptoms bothered them. The reported symptoms were a failing memory (*n* = 33, 65%), difficulties with concentration (*n* = 30, 59%), and experienced jealousy (*n* = 27, 53%) (data not shown). No associations were found between BSA or MADRS scores and the participant’s age, duration of AAS use, time since last AAS use, dose, or self-reported adverse effects.

Lutenizing hormones (LH) regulate the testosterone production in men. As reported earlier in this study cohort, LH concentrations increased from inclusion to the six-month follow-up, suggestive of recovery of the hypothalamic–pituitary axis [17]. There were no correlations between LH or testosterone levels and MADRS or BSA scores, or the separate CPRS items related to aggression, at either the inclusion day or at the follow-up in 19 individuals after six months. Moreover, there were no correlations between the increase in LH and the decrease in MADRS or BSA scores (data not shown).

## 4. Discussion

The novelty of this study is that the psychiatric symptoms associated with AAS use were assessed by different approaches. In addition to direct self-reporting on the inclusion day, SCID and CPRS were conducted by a psychiatrist and a study nurse, respectively, and the CPRS was repeated six months after the subjects stopped using AAS. In addition, all subjects were screened for AAS and drugs of abuse at inclusion, allowing a comparison of self-reported drug intake and urine findings.

The number of individuals that reported aggressive feelings is in line with another study where persons with their own doping experience, as well as their relatives, reported aggression as an adverse effect when contacting the Anti-Doping Hot-Line [2]. Moreover, case reports describe aggression as an adverse effect in AAS users [25]. Controlled studies, however, failed to find an association when supra-physiological doses of AAS were administered to healthy men [26,27]. It has been reported that individuals who are using or intend to use AAS have significantly higher aggressive tendencies prior to use [28,29]. However, in our population, only 9% self-reported aggressive feelings prior to AAS use. The disparity may be due to the fact that Sagoe [29] and Jenssen [28] studied adolescents and their results were based on surveys.

Fifty-two percent of the AAS users subject to SCID evaluation were diagnosed with a PD, with the prevalence being higher than in “normal” populations, where prevalences between 9% and 15% have been reported [30]. Here, we observed that those diagnosed with PD were more likely to report aggressive feelings. Individuals with anti-social or borderline PD have been reported to be predisposed to aggressive or self-destructive behaviors when under stressful conditions, and a relationship between PD and aggression [31], as well as violence and criminality [32], is well-known in non-AAS users. Other studies have found a higher prevalence of psychiatric diagnoses among AAS users than in non-users [10,11]. Whether these psychiatric disorders are primarily induced by AAS or other concomitant drug use or if they are due to pre-existing psychiatric pathology is not known. Considering that SCID, as opposed to CPRS, reflects both current and past symptoms and disorders, the latter is more likely. The use of narcotics was also very common among our study participants, especially among the individuals with a PD diagnosis. It cannot be judged if the aggressive feelings were triggered by AAS as such, by narcotics, or by a combination of both. In conclusion, our findings are in line with previous studies suggesting that PD and the co-use of narcotics may partly explain the association between AAS and aggressive behaviors [33].

Another common symptom self-reported here and elsewhere is depressive feelings [2,7]. In this study, depression was identified by both SCID and CPRS at inclusion. Of the individuals with a SCID I diagnosis of depression, 81% also fulfilled the criteria according to MADRS. Both are clinically used instruments, but SCID also reflects symptoms from the past. The results were well in line with each other. There was a numerical, though not statistically significant, decrease in the MADRS scores from inclusion to the six-month follow-up (Figure 2). It is well-recognized that androgenic steroid-induced hypogonadism is associated with depressive symptoms [7,34]. In this study, 35 individuals (63%) showed testosterone levels below 12 nmol/L at inclusion [35], with levels over 12 nmol/L being considered normal [36]. Moreover, the LH levels in these participants were much lower than among non AAS users [16], so AAS-induced hypogonadism could explain, at least in part, the depressive disorder. However, no correlations between LH and MADRS scores was found, which indicates a complex relationship that is not only related to testosterone deficiency per se. Interestingly, animal studies have reported that AAS-induced reductions in neurotransmitters are associated with anhedonia [37], which is a symptom of depression associated with other recreational drugs [38]. Future studies may include methods to monitor neurochemical changes in the brain (e.g., Positron emission tomography (PET)/ Magnetic resonance imaging (MRI) in relation to AAS cessation.

Sixty-nine percent of the individuals rated high scores (i.e., >11) on the BSA scale of anxiety. Individuals contacting the Anti-Doping Hot-Line often describe concerns about experienced negative effects of AAS [2]. One study described a high self-reported lifetime prevalence of seeking professional expertise for psychiatric problems such as depression and anxiety [39], which is in line with our findings. Six months after inclusion, the BSA scores had decreased significantly. This could be due to the AAS cessation itself or to less concerns about adverse effects. Since AAS use is illegal in Sweden, AAS users may be afraid of legal penalties. This, together with a low trust among AAS users for physicians’ knowledge about AAS [40], may have reinforced the perceived anxiety on the inclusion day. Improving the health care staff´s knowledge about AAS may be an important way to increase AAS users’ trust in the health care and to increase their willingness to seek medical help for adverse effects or for stopping AAS use [40].

A striking result in our study population was the high number of suicidal thoughts and attempts reported. AAS use may be associated with an increased suicide rate [10,41]. The suicide rate may be linked to PD [42], which is a group where a significantly increased risk has been observed [43]. In agreement, the AAS users diagnosed with PD in our population were at a five times increased risk of a (self-reported) suicide attempt and/or suicidal thoughts compared to those without any SCID diagnosis. Moreover, narcotic use and/or other social background factors may partly explain the high rate in our population [42]. Many of the participants reported that they had alcohol or drug abusing parents and that they had a poor relationship with them. In previous studies, it has been noted that AAS users have poorer relationships with their parents [44,45], and it is also known that parents’ substance abuse is a predictor of harmful substance abuse in their off-spring [46]. Individuals who experienced parental divorce as children or adolescents or who had a parent who abused alcohol are at an elevated risk for lifetime suicide attempts [47]. AAS user violence directed at relatives has been reported in one study [45].

This study has limitations that need to be addressed. The recruitment process as such implies a selection bias and the cohort may thus not be representative of AAS users in general, but rather those that contact healthcare providers. Moreover, the number of individuals is low compared to studies based on questionnaire surveys, and the drop-out rate during follow-up was high. Nevertheless, this is, to our knowledge, the only study where SCID and BSA have been studied in verified AAS users. The recruitment process also turned out to be much more difficult than expected. We can only speculate about the possible reasons for this. Firstly, as the use of AAS is illegal in Sweden [48], subjects may have been unwilling to identify themselves and to describe the details of their drug use. Based on our experience, we know that it is a hard-to-reach population, which is consistently seen in other studies as well. Secondly, many may not have been truly interested in stopping AAS use, but rather motivated to participate, in order to get a detailed medical check-up. The latter might also explain the high drop-out rate.

A further limitation is that an oral interview about specific symptoms may overestimate the frequencies of adverse effects, but this study design could also be considered a strength, since the individuals may be more willing to open up, disclose their problems, and provide information that they normally do not intend to report. The co-use of narcotics can be considered a confounder when interpreting the results. Unfortunately, this disintegration will be difficult to overcome since narcotic use is common among AAS users [5,6]. Finally, this study was conducted around 20 years ago. However, as the AAS use pattern appears to be similar in Sweden today [49] (daily communication with AAS users via the Anti-Doping Hot-Line), we believe that the results are still representative, although there is a tendency towards continuous AAS use [49] rather than AAS use in cycles.

## 5. Conclusions

In conclusion, our results support the notion that AAS as such may not induce aggression, depressive feelings, or anxiety. Rather, a combination of AAS, narcotics, and family and medical backgrounds, including a diagnosis of PD, may explain the aggressive feelings/behaviors and suicidal ideation. Many of the psychiatric symptoms as evaluated in CPRS appear to be reversible, which could be of importance when motivating AAS users to stop their use.

This study population will provide a unique opportunity to gain more knowledge about the long-term effects of ongoing and/or previous AAS use, as this remains unstudied. We are planning to conduct a follow-up register study. Such new knowledge would be important for producing a knowledge base for better care, treatment, and prevention of the widespread use of AAS in society.

## Figures and Tables

**Table 1:** Structured Clinical Interview for Diagnosis (SCID) I and II diagnoses in anabolic androgenic steroids (AAS) users.

SCID-I (*n* = 39) Psychiatric Disorders Number of Individuals (%)	SCID-II (*n* = 31) Personality Disorders Number of Individuals (%)
Depressive 16 (41)	Antisocial 10 (32)
Panic 5 (13)	Borderline 6 (19)
Bipolar affective 4 (10)	Paranoid 2 (6)
Delusional 2 (5)	Narcissistic 2 (6)
Post-traumatic stress 1 (3)	Obsessive-compulsive 2 (6)
Obsessive-compulsive 1 (3)	Avoidant 1 (3)

**Table 2:** In the first column, the characteristics of AAS use in all male study subjects are presented. In the two columns to the right, characteristics for the 31 individuals who completed SCID are presented; 16 individuals met the criteria of a personality disorders (PD) diagnosis.

Characteristics	Median (Range) All Individuals *n* = 56	Median (Range) PD Diagnoses *n* = 16	Median (Range) Non-PD Diagnoses *n* = 15
Age at start of AAS use (years)	19.5 (14–39)	19 (15–29)	19 (16–28)
Age at inclusion in the study (years)	25 (15–56)	24 (19–44)	21(18–41)
Duration of AAS use (years)	4 (0.5–16)	5.0 (2–10)	2.0 (1–12)
Duration of AAS cycle (weeks)	8 (4–20)	8 (4–32)	8 (4–21)
Number of AAS (per cycle)	3 (1–5)	3 (1–5)	4 (1–5)
Weekly dose * (mg)	546 (60–3789)	847 (164–3789)	699 (150–3150)
Number of cycles (per year)	2 (1–6)	2 (1–3)	3 (1–4)
Ongoing use of narcotic agents/alcohol	28 (67%)	12 (75%)	5 (33%)

* Regardless of potency.

**Table 3:** AAS substances identified in urine among the 56 study subjects.

AAS Substance	No. of Positive Tests (% of Subjects)
Nandrolone	26 (46%)
Methandrostenolone	8 (14%)
Stanozolol	14 (25%)
Testosterone	20 (36%)
Methenolone	4 (7%)

**Table 4:** The most common accessory medications self-reported by the 56 male study subjects.

Drug	No. of Users (%).
**Doping agents**	
Ephedrine	30 (54)
Clenbuterol	21 (37)
Human choriogonadotropin (hCG)	16 (29)
Clomiphene	7 (12)
Tamoxifen	6 (11)
Prohormone (Precursor)	6 (11)
Growth Hormone (GH)	6 (11)
Insulin	4 (7)
Insulin-like growth factor 1 (IGF-1)	2 (4)
**Dietary supplements**	
Creatine	32 (57)
Protein powder	29 (52)
Amino acids	10 (18)
Caffeine	5 (9)
**Other drugs**	
Sleeping pills	12 (21)
Non-steroidal anti-inflammatory drugs (NSAID)	12 (21)
Benzodiazepines	12 (21)
Sildenafil (Viagra)	6 (11)
Paracetamol	5 (9)
Thyroid hormone (T3)	2 (4)
Isotretinoin	2 (4)
**Narcotic agents**	
Amphetamine	22 (39)
Ecstasy	21 (37)
Cannabis	19 (34)
Cocaine	18 (32)
Opioids	8 (14)
Lysergic acid diethylamide (LSD)	7 (12)
Gamma-Hydroxybutyric acid (GHB)	6 (11)

**Table 5:** The most common psychiatric symptoms and somatic adverse effects reported by 56 male study subjects.

Somatic Adverse Effect	No. of Users (%)	Psychiatric Symptom	No. of Users (%)
Acne	34 (61)	Aggression	40 (71)
Gynecomastia	33 (59)	Depression	30 (54)
Decreased libido	33 (59)	Anxiety	25 (45)
Stretch marks	29 (52)	Violence	25 (45)
Heart problems (arrhythmia and chest pain)	17 (30)	Sleeping disorder	24 (43)
Urination problems	9 (16)	Mood swings	23 (41)
Testicle problems (atrophy and soreness)	9 (16)		

## Supplementary Materials

The following are available online at https://www.mdpi.com/1010-660X/56/6/265/s1.
