# Supplementary material for: Male Anabolic Androgenic Steroid Users with Personality Disorders Report More Aggressive Feelings, Suicidal Thoughts, and Criminality
Source: Medicina (Kaunas). 2020 May 28;56(6):265. doi: 10.3390/medicina56060265 (PMC7353874; doi:10.3390/medicina56060265)
Supplement: Supplementary file 1 [file medicina-56-00265-s001.pdf]

Additional file. Schedule of used AAS-substances, converted to mg per day

Generic name/ trade name n	Nandrolone decanoate (Deca Durabol)	Methandrostenolone (Dianabol, Naposim, Methandienone)	Drostanolone propionate (Masteron)	Oxandrolone (Oxandrolon, Anavar)	4 components testosterone blend (Omnadren)	Oxymetholone (Oxymetholone)	Methenolone (Primobolan)	Testosterone blend (Spectriol)	Testosterone blend (Sustanon)	Testosterone enanthate (Testoviron)	Stanozolol (Winstrol injection)	Stanozolol (Winstrol tablets)	Testosterone undecanoate (Andriol, Restandol testocaps)	Testosterone cypionate (Testosterone Cypionate)	Parabolan (Trenbolone)
1	186.2	64.7		50											
2	14.2								178.5	71.4	14.2				
3	57.1	26.7					25		31.2	14.2					19
4		20								95					
5	33.3		14.2		44.6		17.8				8.3				
6		25													
7													120		
8		150								71.4			320		
9		45													
10	114						14.2				14.2				
11		25					14.2		35.7						
12	Couldn´t declare														
13		40									125				
14									53.5		10.7				
15						50			35.7	35.7					
16	80								35.7	35.7		28.5			
17		60													
18		60			35.7				71.4		14.2				
19	28.5	30							71.4						
20	Couldn't declare														
21		25							142.8		100				
22	33.3						20								
23	40	50					25								
24	57.1	38.8	28.5											71,4	
25		17.8							29.7	29.7					
26	14.2	35							35.7	35.7					
27							28.5				50				
28		20										40			
29	28.5								35.7						
30	50	20		40	62.5						16.6				
31									62.5						
32	250	100				100									
33		80			83.3				83.3		16.6				
34	14.2	25									7.14				
35		25				50				35.7	7.14				
36	28.5	20								35.7	14.2				
37	7.14				17.8				17.8						
38	28.5	50			107					142.8			260		
39	85.7	50													
40											21.4				
41	57.1						28.5				14.2				
42	57.1				390										
43	57.1									35.7					
44											16.6				
45		35													
46								32.5			50				
47	57.1	25					107				14.2				
48	Couldn't declare														
49								9.2			14.2				
50	28.5								35.7	35.7					
51	28.5			10	50						14.2				
52	Couldn't declare														
53														28.5	
54	Couldn´t declare														
55			60												
56	71.4	50							71.4						
